# Real-Time Accumulative Computation Motion Detectors

**DOI:** 10.3390/s91210044

**Published:** 2009-12-10

**Authors:** Antonio Fernández-Caballero, María Teresa López, José Carlos Castillo, Saturnino Maldonado-Bascón

**Affiliations:** 1 Instituto de Investigación en Informática de Albacete, 02071-Albacete, Spain; E-Mails: mlopez@dsi.uclm.es (M.T.L.); josecarlos@dsi.uclm.es (J.C.C.); 2 Departamento de Sistemas Informáticos, Escuela de Ingeníeros Industrials de Albacete, Universidad de Castilla-La Mancha, 02071-Albacete, Spain; 3 Departamento de Sistemas Informáticos, Escuela Superior de Ingeniería Informática, Universidad de Castilla-La Mancha, 02071-Albacete, Spain; 4 Department of Signal Theory and Communications, Escuela Politécnica Superior, Universidad de Alcalá, 28871-Alcalá de Henares, Madrid, Spain; E-Mail: saturnino.maldonado@uah.es

**Keywords:** accumulative computation, finite state automata, real-time, motion detection

## Abstract

The neurally inspired accumulative computation (AC) method and its application to motion detection have been introduced in the past years. This paper revisits the fact that many researchers have explored the relationship between neural networks and finite state machines. Indeed, finite state machines constitute the best characterized computational model, whereas artificial neural networks have become a very successful tool for modeling and problem solving. The article shows how to reach real-time performance after using a model described as a finite state machine. This paper introduces two steps towards that direction: (a) A simplification of the general AC method is performed by formally transforming it into a finite state machine. (b) A hardware implementation in FPGA of such a designed AC module, as well as an 8-AC motion detector, providing promising performance results. We also offer two case studies of the use of AC motion detectors in surveillance applications, namely infrared-based people segmentation and color-based people tracking, respectively.

## 1. Introduction

Motion analysis in image sequences is a constantly growing discipline due to the great number of applications in which it plays a primordial key function. Moreover, optical flow in monocular video can serve as a key for recognizing and tracking moving objects, as flow data contains richer information and in experiments can successfully track difficult sequences [1]. In this sense, recently some approaches have used optical-flow processing systems to analyze motion in video sequences in real-time [2, 3]. Some outstanding approaches to motion detection are biologically (neurally) inspired (e.g., [4–8]). Also in the last few years, the neurally inspired accumulative computation (AC) method [9–12] and its application to motion detection have been introduced [13–15]. Currently our research team is involved in implementing the method into real-time in order to provide efficient performance in visual surveillance applications [16–18].

In this sense, many researchers have explored the relation between discrete-time neural networks and finite state machines, either by showing their computational equivalence or by training them to perform as finite state recognizers from example [19]. The relationship between discrete-time neural networks and finite state machines has very deep roots [20–22]. The early papers mentioned show the equivalence of these neural networks with threshold linear units, having step-like transfer functions, and some classes of finite state machines. More recently, some researchers have studied the close relationships more in detail [23, 24], as well as the combination of connectionist and finite state models into hybrid techniques [25, 26]. From the excellent survey on the work by [24] that has established a connection between finite state machines and neural networks, we highlight some predominant ideas. Firstly, consider that finite state machines constitute the best characterized computational model, whereas artificial neural networks have become a very successful tool for modeling and problem solving. And indeed, the fields of neural networks and finite state computation started simultaneously. A McCulloch-Pitts net [20] really is a finite state of interconnected McCulloch-Pitts neurons. Kleene [21] formalized the sets of input sequences that led a McCulloch-Pitts network to a given state, and later, Minsky [22] showed that any finite state machine can be simulated by a discrete-time neural net using McCulloch-Pitts units. During the last decades specialized algorithms even have extracted finite state machines from the dynamics of discrete-time neural networks [27–30]. Now, also consider the fact that the use of neural networks for sequence processing tasks has a very important advantage: neural networks are adaptive and may be trained to perform sequence processing tasks from examples. An important issue in the motivation of this paper is that the performance of neural networks—especially during learning phase—can be enhanced by encoding a priori knowledge about the problem directly into the networks [31, 32]. This knowledge can be encoded into a neural network by means of finite state automata rules [33].

Our experience up to date has shown that most applications in computer vision, and more specifically in motion detection through AC, offer good results with the same values of the parameters of the model. The article shows how to reach real-time performance after using a model described as a finite state machine. The two steps towards that direction are: (a) A simplification of the general AC method is performed by formally transforming it into a finite state machine. (b) A hardware implementation of such a designed AC module, as well as an 8-AC motion detector, providing promising performance results. The rest of the paper is structured as follows. Section 2. revisits the AC method in motion detection. Then, section 3. introduces the simplified model for AC in form of a finite state automaton. Section 4. depicts the real-time hardware implementation of motion-detection AC modules obtained from the previous formal model. Lastly, 5. and 6. are the Data and results and Conclusions sections, respectively.

## 2. Accumulative Computation (AC) in Motion Detection

### 2.1. Classical Motion Detection Approaches

The two main problems in motion analysis in image sequences are the correspondence and the aperture problem. The correspondence problem, well exposed by Duda and Hart [34], is related to the relation velocity-sampling rate, and defines two broad research lines. The first one consists in studying two consecutive images in a static manner and then analyzing how some significant pixels have moved between both frames. The second line consists in locally studying each pixel and its neighborhood along time. The aperture problem, also broadly treated [35–40] is related to the task of associating the apparent movement in the environment of a concrete pixel with the real movement of the element to which this pixel belongs. The complexity of the problem increases in three-dimensional scenes [41].

Models based on local motion detection face the correspondence problem considering that a pixel in time *t* + Δ*t* is close to the same pixel in time instant *t*. These models are usually based on gradient analysis or local correlation. Some gradient analysis models calculate the velocity using the spatial-temporal derivative of the brightness in a pixel and its immediate environment. Among this type of models we can highlight the direction selectivity model of Marr and Ullman [37], which obtains the direction of motion but not the velocity. Lawton's motion direction prediction model [42] calculates the direction of the velocity from the gradient. Fennema and Thompson [35] calculate the velocity using the gradient, but they impose restrictions on velocity and gray level. The most extended model of this family is the optical flow, proposed by Horn and Schunck [36], which calculates the apparent velocity of each pixel using the spatial and temporal gradient of the brightness in each pixel. This model imposes the uniformity constraint, and the non-existence of spatial discontinuities in the shapes.

Correlation based models [43, 44] are usually based on correlating the brightness of a pixel and its closer neighbors along time. Some of them are the relational selectivity model of Reichardt and Hassenstein [43] or the direction selectivity model of Barlow and Levick [44], which calculate the direction of velocity by comparing the input value with the previous one and with the neighbors. Another group of this type of models is based on spatio-temporal energy [45, 46]. In Heeger's model [46] image sequences are represented as a three-dimensional space, two spatial and one temporal, which calculates the velocity by means of three-dimensional filters. The model of human vision of Watson and Ahumada [47] is correlational but uses biologically inspired tools.

There are also models based on the uniformity restriction. These impose that the moving objects velocity fields vary uniformly, since objects usually have uniform surfaces. They analyze local velocity fields to obtain information about the real velocity of the objects. Some examples are the visual motion measurement model of Hildreth [39], the neural networks primary vision model of Koch, Marroquin and Yuille [48], and, the model of computational theory for the perception of the coherent visual motion of Yuille and Grzywacz [49].

### 2.2. Description of Accumulative Computation

The method proposed, based on the effect called permanency [50], is performed on those sensor pixels where motion is detected during a time interval [*t* − Δ*t, t*], where Δ*t* is the maximum time between the total discharge and the saturation associated to each pixel of the input image sensor. The concept of permanency, associated to pixel (*i, j*) is related to the time elapsed with no variation in the image input signal *I*(*i, j; t*) on this pixel. The variable associated to the permanency concept is defined as the accumulative computation charge. This is the main difference of our method compared to other motion analysis methods related to the optical flow In other approaches, the analysis is only performed on image pixels where motion has taken place in the present time *t*.

The AC approach is neurally inspired. Usually the time evolution of the neuron membrane potential is modeled by a first order differential equation known as the “leaky integrator model”. A different way of modeling time evolution of membrane potential is to consider the membrane as a local working memory in which neither the triggering conditions nor the way in which the potential tries to return to its input-free equilibrium value, needs to be restricted to thresholds and exponential increases and decays. This type of working memory is characterized by the possibility of controlling its charge and discharge dynamics in terms of:

1. The presence of specific spatio-temporal features with values over a certain threshold.
2. The persistency in the presence of these features.
3. The increment or decrement values (±*δQ*) in the accumulated state of activity of each feature and the corresponding current value, *Q*(*t*).
4. The control and learning mechanisms.

**Figure 1.**
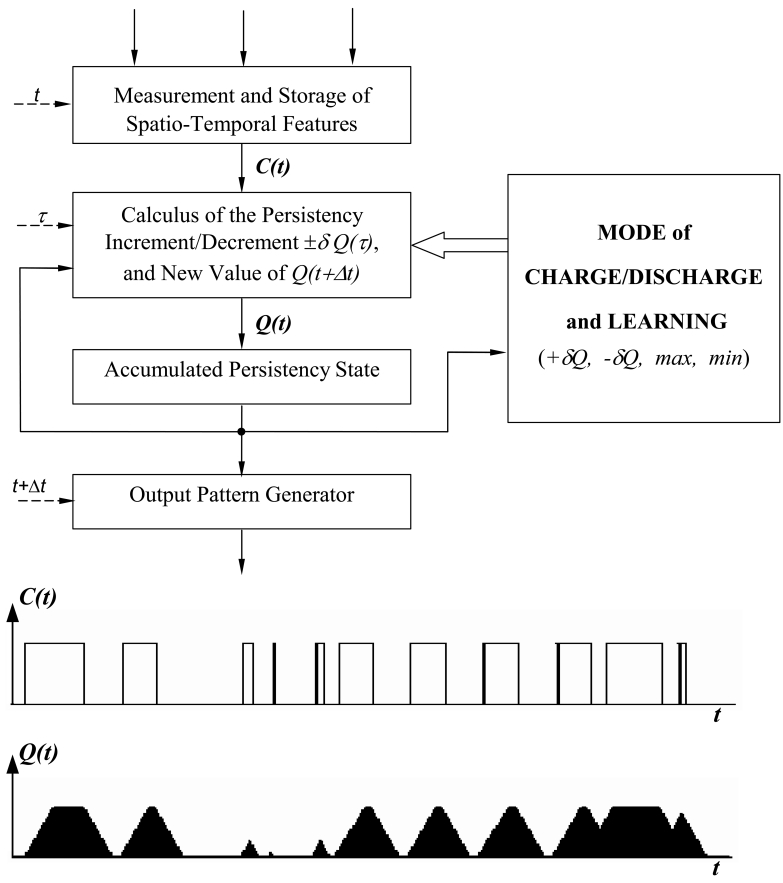
The AC working memory model (upper part) and an example of the temporal evolution of the accumulated persistency state, *Q*(*t*), in response to a specific sequence of input values (lower part).

The upper part of Figure 1 shows the AC model's block diagram. The lower part of Figure 1 illustrates the temporal evolution of the state of the charge in an AC working memory in front of a particular one-dimensional stimuli sequence. From [9, 10] we reformulate the equations of the AC method as formulated for the motion detection task. Firstly, Equation (1) covers the need to segment each input image *I* into a preset group of gray level bands (*N*).

(1) $$I_{k}(i,j;t)=\{\begin{array}{l} 1,\;\text{if}\;I(i,j;t)\in \left[\frac{256}{N}\cdot k,\frac{256}{N}\cdot (k+1)-1\right]\\ 0,\;\text{otherwise} \end{array}$$

This formula assigns pixel (*i, j*) to gray level band *k*. Then, the accumulated charge value related to motion detection at each input image pixel is obtained, as shown in formula (2):

(2) $$Q_{k}(i,j;t)=\{\begin{array}{ll} \min & \text{if}\;{\text{I}}_{k}(i,j;t)=0\\ \max & \text{if}\;({\text{I}}_{k}(i,j;t)=1)\;\text{AND}\;({\text{I}}_{k}(i,j;t-\Delta t)=0)\\ \max[Q_{k}(i,j;t-\Delta t)-\delta Q,\min] & \text{if}\;(I_{k}(i,j;t)=1)\;\text{AND}\;(I_{k}(i,j;t-\Delta t)=1) \end{array}$$

The charge value at pixel (*i, j*) is discharged down to *min* when no motion information is available, is saturated to *max* when motion is detected at *t*, and, is decremented by a value δ*Q* when motion goes on being detected in consecutive intervals *t* and *t* − Δ*t*.

## 3. Simplified Model for AC in Motion Detection

The control knowledge is described extensively by means of a finite automaton in which the state space is constituted from the set of distinguishable situations in the state of accumulated charge in a local memory [11]. Thus, we distinguish *N* + 1 states *S*_0_, *S*_1_, …, *S_N_*, where *S*_0_ is the state corresponding to the totally discharged local memory (*min*; in general *min* = 0), *S_N_* is the state of complete charge and the rest are the *N* − 1 intermediate charge states (*S_int_*) between *min* and *max*.

### 3.1. Initial Model

Let us suppose, without loss of generality, that it is enough to distinguish eight levels of accumulated charge (*N* = 8) and, consequently, that we can use as a model of the control underlying the inferential scheme that describes the data flow corresponding to the calculation of this subtask an 8 states automaton (*S*_0_, *S*_1_, …, *S*_7_), where *S*_0_ corresponds to *min* and *S*_7_ to *max*. Let us also suppose that discharge (*δQ* = 1) takes the values corresponding to the descent of one state.

**Figure 2.**
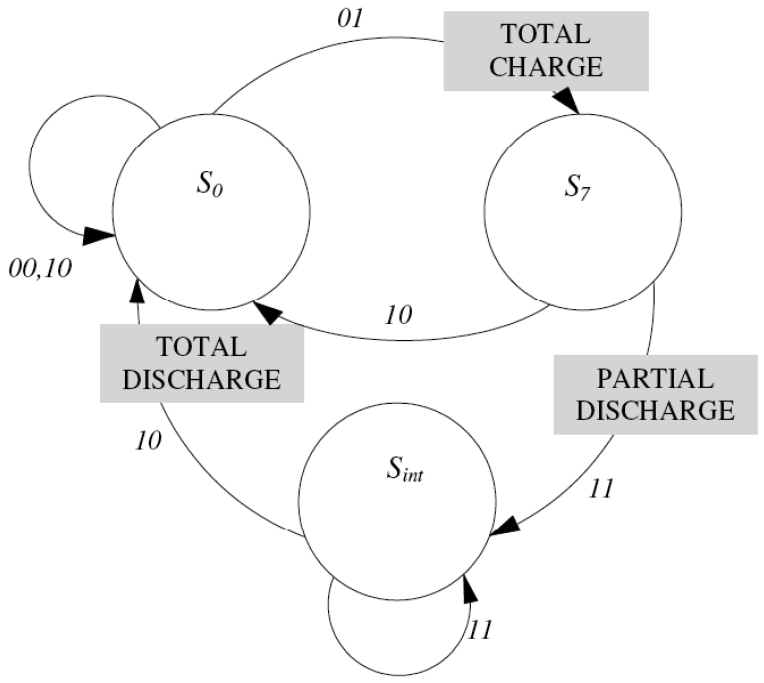
Control automaton that receives inputs *I_k_*(*i, j; t* − Δ*t*) and *I_k_*(*i, j; t*), and produces three outputs, coincident with its three distinguishable charge states (*S*_0_ = *min, S*_7_ = *max*, and *S_int_*).

Now, the aim is to detect the temporal and local (pixel to pixel) contrasts of pairs of consecutive binarised images at gray level *k*. The subtask firstly gets as input data the values of the 256 gray level input pixels and generates *N* = 8 binary images, *I_k_*(*i, j; t*). The output space has a FIFO memory structure with two levels, one for the current value and another one for the previous instant value. Thus, for *N* bands, there are 2*N* = 16 binary values for each input pixel; at each band there is the current value *I_k_*(*i, j; t*) and the previous value *I_k_*(*i, j; t* − Δ*t*), such that Equation (1) turns into:

(3) $$I_{k}(i,j;t)=\{\begin{array}{l} 1,\;\text{if}\;I(i,j;t)\in \left[32\cdot k,32\cdot (k+1)-1\right]\\ 0,\;\text{otherwise} \end{array}$$

where *k* = 0, 1, …, 7, is the band index. Thus, we are in front of a vector quantization (scalar quantization) algorithm generally called multilevel thresholding. As well as segmentation in two gray level bands is a usual thing, here we are in front of a refinement to the segmentation in *N* = 8 gray level bands. Thus, multilevel thresholding is a process that segments a gray-level image into several distinct regions. Figure 2 shows the state transition diagram corresponding to the different inputs and outputs. The following situations can be observed:

1. *I_k_*(*i, j; t* − Δ*t*) = {0, 1}, *I_k_*(*i, j; t*) = 0
  In this case the calculation element (*i, j*) is not able to detect any contrast with respect to the input of a moving object in that band (*I_k_*(*i, j; t*) = 0). It may have detected it (or not) in the previous interval (*I_k_*(*i, j; t* − Δ*t*) = 1, *I_k_*(*i, j; t*) = 0). In any case, the element passes to state S_0_, the state of complete discharge, independently of which was the initial state.
2. *I_k_*(*i, j; t* − Δ*t*) = 0, *I_k_*(*i, j; t*) = 1
  The calculation element has detected in *t* a contrast in its band (*I_k_*(*i, j; t*) = 1), and it did not in the previous interval (*I_k_*(*i, j; t* − Δ*t*) = 0). It passes to state S_7_, the state of total charge, independently of which was the previous state.
3. *I_k_*(*i, j; t* − Δ*t*) = 1, *I_k_*(*i, j; t*) = 1
  The calculation element has detected the presence of an object in its band (*I_k_*(*i, j; t*) = 1), and it had also detected it in the previous interval (*I_k_*(*i, j; t* − Δ*t*) = 1). In this case, it diminishes its charge value in a certain value, *δQ*. This discharge - partial discharge - can proceed from an initial state of saturation *S*_7_, or from some intermediate state (*S*_6_, …, *S*_1_). This partial discharge due to the persistence of the object in that position and in that band, is described by means of a transition from *S*_7_ to an intermediate state, *S_int_*, without arriving to the discharge, *S*_0_. The descent in the element's state is equivalent to the descent in the pixel's charge, as you may appreciate on Figure 2.

### 3.2. Hysteresis Bands

The presented scheme suffers from low performance when a pixel is in the border of two bands. In this situation, a pixel with a mean value in the border of two bands and some noise that makes the pixel change from one band to another close band, activates the stimuli sequence and, consequently, motion is detected when there is no real motion in the scene.

However the scheme can be slightly modified to overcome this problem. Indeed, the previous scheme can be modified to take into account a hysteresis cycle defined through $I_{k}^{+th}$ and $I_{k}^{-th}$.

(4) $$I_{k}^{+th}(i,j;t)=\{\begin{array}{l} 1,\;\text{if}\;I(i,j;t)\in \left[\frac{256}{N}\cdot k+th,\frac{256}{N}\cdot (k+1)-1+th\right]\\ 0,\;\text{otherwise} \end{array}$$

(5) $${\text{I}}_{k}^{-\text{t}h}(i,j;t)=\{\begin{array}{l} 1,\;\text{if}\;I(i,j;t)\in \left[\frac{256}{N}\cdot k-th,\frac{256}{N}\cdot (k+1)-1-th\right]\\ 0,\;\text{otherwise} \end{array}$$

In this case the accumulated charge for band *k* is now rewritten as:

(6) $$Q_{k}(i,j;t)=\{\begin{array}{ll} \min & \;\text{if}\;(I_{k}(i,j;t)=0)\;\text{AND}\;(I_{k}(i,j;t-\Delta t)=0)\\ \max & \;\text{if}\;(I_{k}(i,j;t)=1)\;\text{AND}\;(I_{k}(i,j;t-\Delta t)=0)\;\text{AND}\;(I_{k}^{+th}(i,j;t-\Delta t)=0)\;\text{AND}\;(I_{k}^{-th}(i,j;t-\Delta t)=0)\\ \max[Q_{k}(i,j;t-\Delta t)-\delta Q,\min] & \;\text{if}\;(I_{k}(i,j;t)=1)\;\text{AND}\;(I_{k}(i,j;t-\Delta t)=0)\;\text{AND}\;(I_{k}^{+th}(i,j;t-\Delta t)=1)\;\text{OR}\;(I_{k}^{-th}(i,j;t-\Delta t)=1)\\ \max & \;\text{if}\;(I_{k}(i,j,t)=0)\;\text{AND}\;(I_{k}(i,j,t-\Delta t)=1)\;\text{AND}\;(I_{k}^{+th}(i,j;t)=0)\;\text{AND}\;(I_{k}^{-th}(i,j;t)=0)\\ \max[Q_{k}(i,j;t-\Delta t)-\delta Q,\min] & \;\text{if}\;(I_{k}(i,j;t)=0)\;\text{AND}\;(I_{k}(i,j;t-\Delta t)=1)\;\text{AND}\;(I_{k}^{+th}(i,j,t)=1)\;\text{OR}\;(I_{k}^{-th}(i,j;t)=1)\\ \max[Q_{k}(i,j;t-\Delta t)-\delta Q,\min] & \;\text{if}\;(I_{k}(i,j;t)=1)\;\text{AND}\;(I_{k}(i,j;t-\Delta t)=1) \end{array}$$

where the parameter *th* selects the hysteresis cycle and allows variations in the interval [*v* + *th, v* − *th*] not to be considered as motion. *th* must be selected according to the noise of the images.

## 4. Real-time Hardware Implementation of Motion-Detection AC Modules

In order to accelerate their performance, and hence to obtain real-time processing rates, many applications use reconfigurable hardware. More concretely, they are programmed on field programmable gate arrays (FPGAs) [51, 52]. For instance, the application proposed by Bensaali and Amira [51] is accelerating the color space conversion between *Y'CrCb* and *RGB* color spaces. In [52] an implementation of genetic algorithms in FPGA is proposed.

Some of the most recently used FPGA families are Xilinx Virtex-II [53-55] and Virtex-E [56, 57]. [53] introduces VLSI architectures for the forward 4 × 4 integer approximation of the DCT transform, the 4 × 4 (and 2 × 2) Hadamard transform and quantization that is used as a second level in the transformation hierarchy. In the paper by Moon and Sedaghat [54], a hardware implementation of an adaptive digital pre-distortion system for radio-over-fiber links is described. [55] describes an FPGA device for cryptanalysis of a pseudorandom generator that consists of a number of subgenerators. Damaj [56] explores the effectiveness and extends a formal methodology in the design of massively parallel algorithms. Lastly, [57] presents a new fully reconfigurable 2D convolver designed for FPGA-based image and video processors.

We also highlight a recent paper [58] that presents the implementation of a segmentation process to extract the moving objects from image sequence taken from a static camera used for real time vision tasks. The authors use the low cost Spartan-II device.

**Figure 3.**
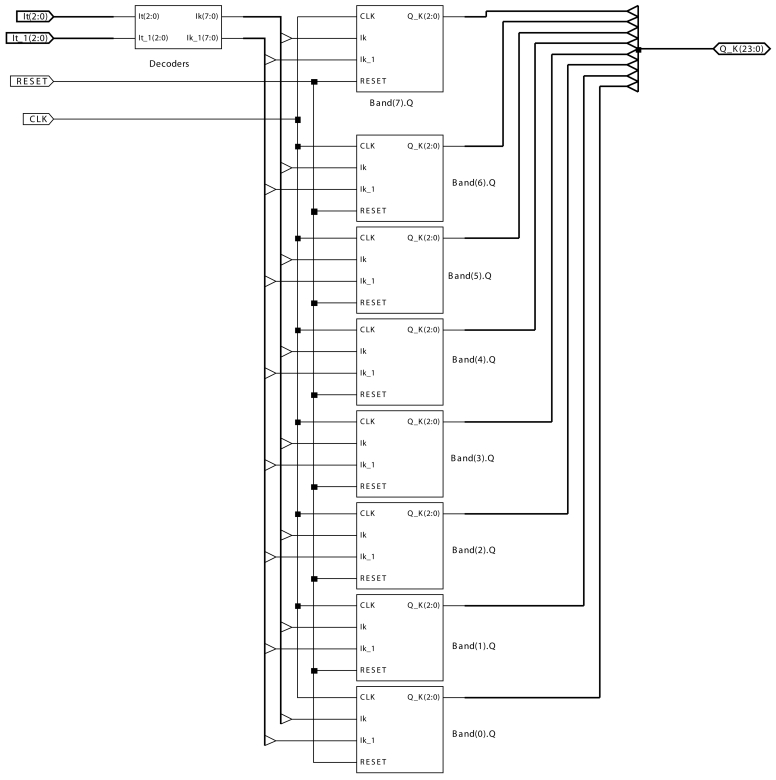
Layout of a motion-detection AC module.

**Figure 4.**
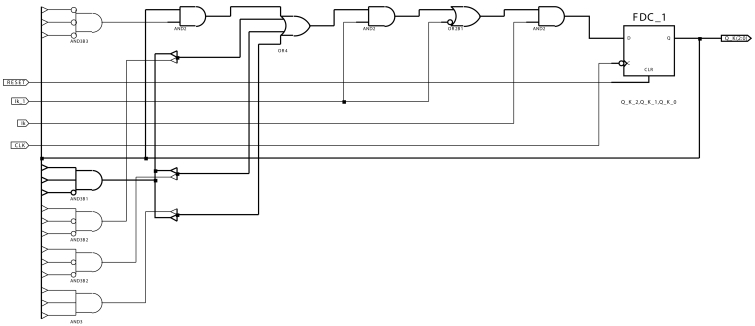
AC module automata for band 7.

**Figure 5.**
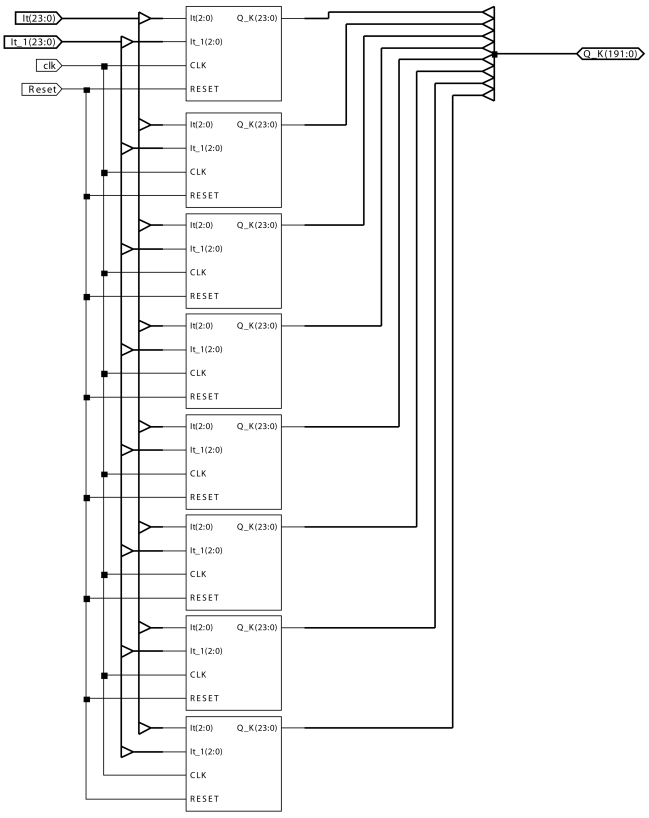
Layout of an 8-AC motion detector.

In this section, we show how a single AC module, as well as its expansion to an 8-module, starting from the description as a finite state machine, has been implemented (see Figures 3 and 4 for the single AC module, and Figure 5 for the 8-AC module, respectively). In order to implement the module, the programming has been performed under Very High Speed Integrated Circuit Hardware Description Language (VHDL), and by means of the Xilinx ISE 10.1 tool, the module has been synthesized and implemented in a Xilinx Virtex-5 FPGA. More concretely, the device used is a 5vfx30tff665-1.

In Table 1, the temporal results associated to the implementation are shown, and in Table 2, the device utilization summary is offered.

Figure 3 shows the layout of a motion-detection AC module. The inputs to the AC module are:

- *It* is the input value at each pixel at time instant *t*.
- *It*_1 is the input value at each pixel at time instant *t* − Δ*t*.
- *CLK* is the clock signal to control the automata associated to the AC module.
- *RESET* is the signal to reset the AC module.

The output *Q_k* is formed by the 24 bits of the charge values corresponding to the 8 bands (3 bits per band).

The same Figure 3 includes a series of blocks. There is a block called *Decoders* and 8 *Band*(*k*)_*Q* blocks associated to the 8 bands. The block *Decoders*, composed by 2 decoders, has as inputs 3 bits corresponding to the input at time instant *t* and 3 bits corresponding to the input at time instant *t* − Δ*t*. The output of this block is an 8 bit vector, where a bit value of 1 is assigned to the position corresponding to band *k*. The rest of the bits take a value of 0. For instance, if the input to *Decoders* is 101, the output will be 00100000.

Each one of the 8 *Band*(*k*)_*Q* blocks includes the necessary combinational and sequential part for implementing each band's proper automata. As an example, Figure 4 shows the implementation for the automata *Band*(7)_*Q*. The rest of the blocks are very similar.

Now, for the implementation of an 8-module, using the same FPGA (the 5vfx30tff665-1), the results obtained are shown in Tables 3 and 4. Notice that each one of the blocks depicted is one AC module as shown in Figure 3.

As the maximum combinational path delay is 4.348 ns, when working with 648 × 480 pixel images, which need 38880 8-AC modules, the results are obtained after 0.167 ms. This performance has to be considered as excellent, enabling working at real-time.

## 5. Data and Results

In order to validate the usefulness of the AC modules described previously, a couple of case studies of the use of AC motion detectors in surveillance applications, namely infrared-based people segmentation and color-based people tracking, respectively, are introduced in this section. The cases introduced only show a few of many possible uses of our approach.

### 5.1. Infrared-Based People Segmentation

**Figure 6.**
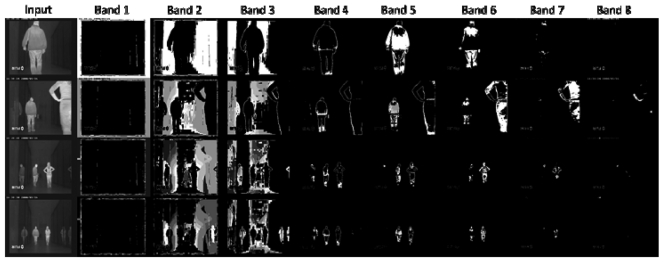
Result of AC detection modules for each gray level band.

We have used an infrared surveillance sequence captured by our research team, where different persons appear and disappear in the scene. Figure 6 shows the result of the AC detection modules dedicated to one of the eight infrared grey level bands.

**Figure 7.**
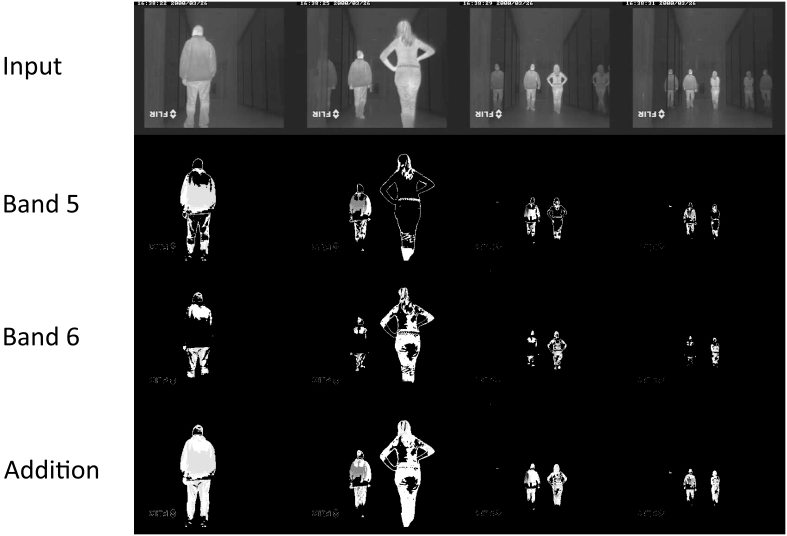
Addition of AC detection modules corresponding to bands 5 and 6 for efficient infrared-based people segmentation.

Notice that motion not detected in one band is detected in another one. Notice that the background motion is mainly obtained at bands 2 and 3, whereas the foreground is obtained at bands 4 to 7. Bands 1 and 7 do not offer much information, neither on foreground nor on background motion. A deeper insight into the figure show some interesting results. A gross conclusion is that band 4 mostly gets the contours of the foreground moving elements (people, in this case), whereas bands 5 and 6 show the main parts of the moving bodies. This is why, in this particular case, it seems reasonable to sum up bands 5 and 6 to obtain moving people in infrared imagery. Now, Figure 7 shows the efficiency of the combination of the AC modules corresponding to bands 5 and 6 for segmenting moving people in the sequence.

### 5.2. Color-Based People Tracking

**Figure 8.**
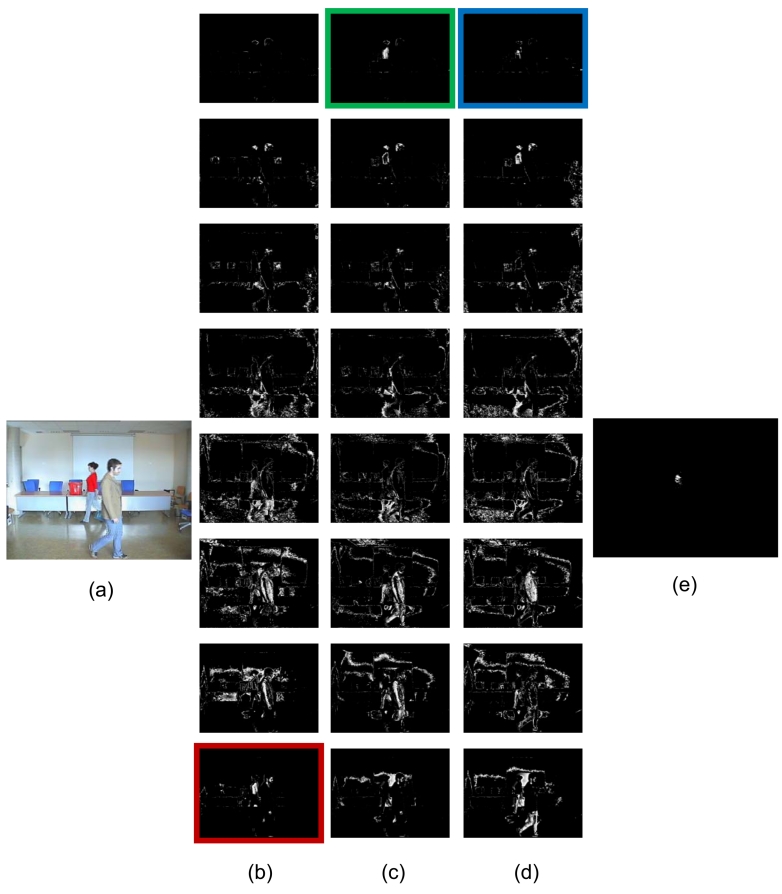
Result of AC detection modules for color-based people tracking. (a) Input image. From top to bottom, bands 0 to 7, result of AC on the (b) *R* component, (c) *G* component, (d) *B* component. (e) Result of tracking the red t-shirt.

In this case study, we have used a data set containing 1109 frames captured in an office room. Figure 8a offers one input image number of the sequence. Here, for the purpose of testing the proposal applied to color images, we are interested in tracking a range of colors in the *RGB* (reg-green-blue) color model. This range has to cover in this case a red t-shirt dressed by a young woman. This could be a typical example of tracking suspicious people in the visual surveillance domain.

#### Simple tracking algorithm

As you may appreciate in Figure 8b, c and d, none of the AC modules dedicated to the eight bands for the *R, G*, or *B* components, respectively, is capable of segmentating/tracking the range of colors selected. Moreover, you may appreciate that there is a lot of noise in the images provided. Here, in order to obtain the final result of Figure 8e, some logical operations were necessary. We multiplied (logical AND) the result of band 7 for the *R* component and the the result of band 0 for the *G* y *B* components.

**Figure 9.**
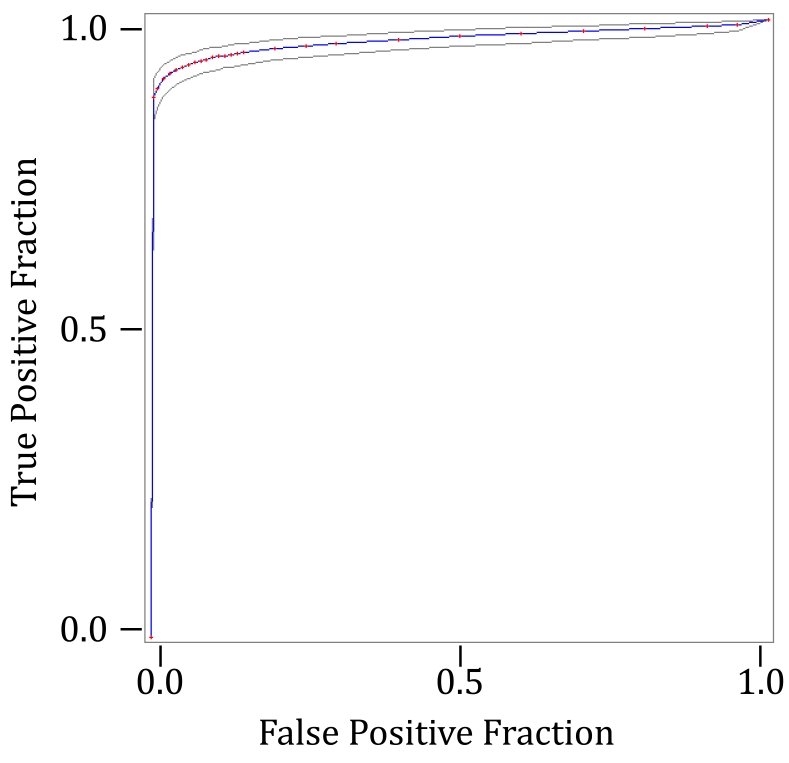
ROC curve associated to the color video sequence.

Table 5 shows some statistics about the performance of the algorithm as applied to the complete input video sequence. Also, Figure 9 shows the ROC curve associated. At a first glance, you may observe that the curve grows very quickly and is very close to the maximum value of 1. The area under the curve (see Table 5, “Empiric ROC Area”) is 0.964, which clearly states that our method throws excellent results. The area calculates the method's ability to discriminate between detected and not detected objects.

#### Enhanced tracking algorithm

The sequence has been analyzed using the hysteresis modification proposed with different settings. In this case the charge of all bands $Q_{k}^{i}$, where *i* ∈ {*R, G, B*} and *k* = 0, …, *N_i_*−1, have been added to obtain a total charge *Q_T_*. The algorithm shows promising results for detecting motion with low complexity The number of bands *N_i_* and the threshold *th_i_* must be selected according to the noise in the image.

**Figure 10.**
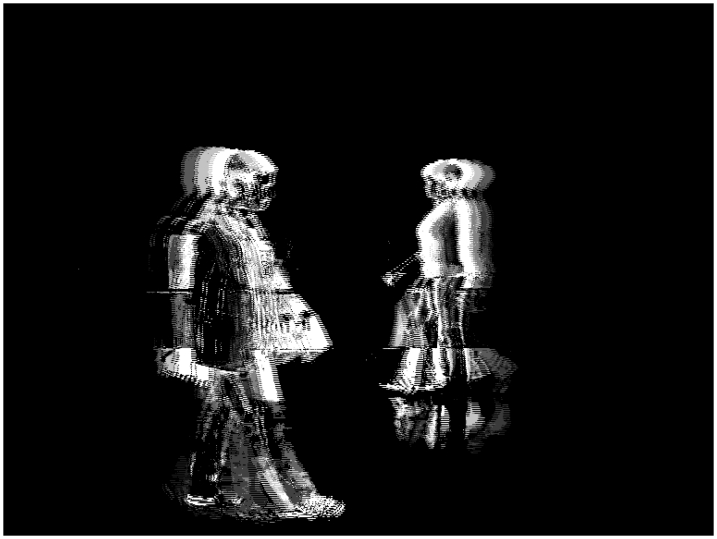
Total charge for *N_R_* = 4, *N_G_* = 4, *N_B_* = 4 and *th_R_* = 45, *th_G_* = 45 and *th_B_* = 45.

**Figure 11.**
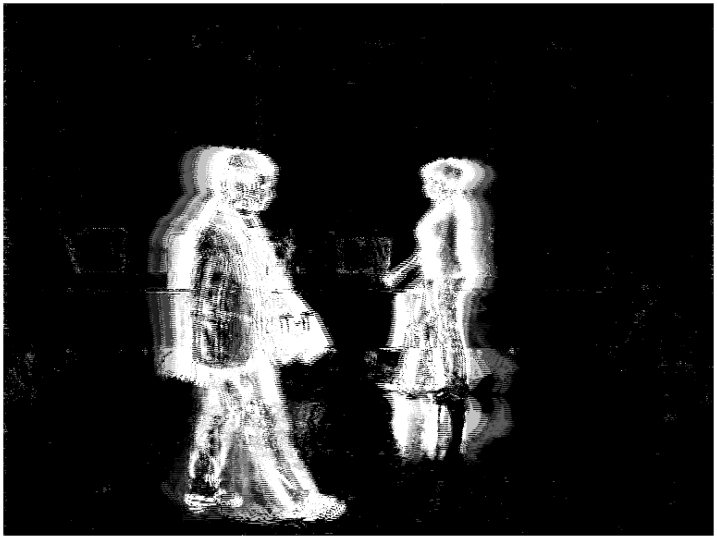
Total charge for *N_R_* = 8, *N_G_* = 8, *N_B_* = 8 and *th_R_* = 16, *th_G_* = 16 and *th_B_* = 16.

**Figure 12.**
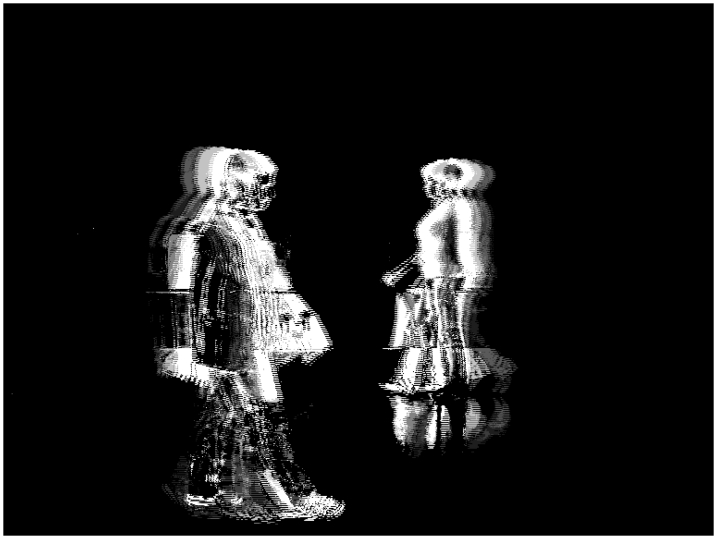
Total charge for *N_R_* = 4, *N_G_* = 8, *N_B_* = 8 and *th_R_* = 60, *th_G_* = 30 and *th_B_* = 30.

Figure 10 shows the total charge *Q_T_* for *N_R_* = 4, *N_G_* = 4, *N_B_* = 4 and *th_R_* = 45, *th_G_* = 45 and *th_B_* = 45. Also, Figure 11 shows the total charge *Q_T_* for *N_R_* = 8, *N_G_* = 8, *N_B_* = 8 and *th_R_* = 16, *th_G_* = 16 and *th_B_* = 16. Lastly, Figure 12 shows the total charge *Q_T_* for *N_R_* = 4, *N_G_* = 8, *N_B_* = 8 and *th_R_* = 60, *th_G_* = 16 and *th_B_* = 16.

From the results offered it can be easily seen that, when the number of bands *N_i_* is increased and the *th_i_* is decreased, the noise in *Q_T_* is incremented. In the opposite case, noise is reduced, but some of the moving objets are not detected. Thus, parameters *N_i_* and *th_i_* must be selected as a trade off between both situations.

## 6. Conclusions

This paper starts from previous works in computer vision, where our accumulative computation method applied to motion detection has proven to be quite efficient. We have shown in this article how the AC model, based in neural networks, has been modeled by means of finite state automata, seeking for real-time through an implementation in FPGA-based reconfigurable hardware. Therefore, two steps towards that direction have been taken: (a) A simplification of the general AC method by formally transforming it into a finite state machine. (b) A hardware implementation of such AC modules.

The design by means of programmable logic enables the systematic and efficient crossing from the descriptions of the functional specifications of a sequential system to the equivalent description in terms of a finite state automaton. Starting from this point, a hardware implementation by means of programmable logic is very easy to perform. This kind of design is especially interesting in those application domains where the response time is crucial (e.g., monitoring and diagnosing tasks in visual surveillance and security).

In this paper, the results obtained after implementing AC modules in hardware on programmable logic, concretely on Virtex-5 FPGA's, have been shown. These results start from previous validated researches on moving objects detection, which unfortunately did not reach real-time performance. Prior to the implementation, a simplification of the model into an 8-state finite automaton has been performed. The procedure is easily expandable to all delimited-complexity functions that may be described in a clear and precise manner by a not too high number of states.

Two case studies of real interest in surveillance applications have been introduced. These examples have demonstrated the versatility of the motion detectors, which can be inserted into any high-level computer vision task.

## Figures and Tables

**Table 1.** Temporal results for the AC module.

Minimum period	1.287 ns
Maximum frequency	777.001 MHz
Minimum input required time before clock	2.738 ns
Maximum output delay after clock	3.271 ns

**Table 2.** Device utilization summary for the AC module.

Slice Logic Utilization:	
Number of Slice Registers	24 out of 20480 (0%)
Number of Slice LUTs	40 out of 20480 (0%)
Number used as Logic	40 out of 20480 (0%)
Slice Logic Distribution:	
Number of LUT Flip Flop pairs used	40
Number with an unused Flip Flop	16 out of 40 (40%)
Number with an unused LUT	0 out of 40 (0%)
Number of fully used LUT-FF pairs	24 out of 40 (60%)
Number of unique control sets	1
IO Utilization:	
Number of IOs	32
Number of bonded IOBs	32 out of 360

**Table 3.** Temporal results for the 8-AC motion detector.

Minimum period	2.736 ns
Maximum frequency	365.497 MHz
Minimum input required time before clock	2.834 ns
Maximum output delay after clock	3.271 ns
Maximum combinational path delay	4.348 ns

**Table 4.** Device utilization summary for the 8-AC motion detector.

Slice Logic Utilization:	
Number of Slice Registers	248 out of 20480 (1%)
Number of Slice LUTs	467 out of 20480 (2%)
Number used as Logic	467 out of 20480 (2%)
Slice Logic Distribution:	
Number of LUT Flip Flop pairs used	492
Number with an unused Flip Flop	244 out of 492 (49%)
Number with an unused LUT	25 out of 492 (5%)
Number of fully used LUT-FF pairs	223 out of 492 (45%)
Number of unique control sets	2
IO Utilization:	
Number of IOs	260
Number of bonded IOBs	260 out of 360 (72%)
Number of BUFG/BUFGCTRLs	1 out of 32 (3%)

**Table 5.** Algorithm performance statistics for the color video sequence.

Number of Cases:	1109
Number of Correct Cases:	1066
Accuracy:	96.1%
Sensitivity:	95.8%
Specificity:	96.4%
Positive Cases Missed:	24
Negative Cases Missed:	19

Fitted ROC Area:	0.968
Empiric ROC Area:	0.964
